# Thermal and Hydrothermal Treatment of Silica Gels as Solid Stationary Phases in Gas Chromatography

**DOI:** 10.1155/2013/931067

**Published:** 2013-09-25

**Authors:** Ashraf Yehia El-Naggar

**Affiliations:** ^1^Chemistry Department, Faculty of Science, Taif University, Al-Haweiah, P.O. Box 888, Taif 21974, Saudi Arabia; ^2^Egyptian Petroleum Research Institute, Nasr City, Cairo, Egypt

## Abstract

Silica gel was prepared and treated thermally and hydrothermally and was characterized as solid stationary phase in gas chromatography. The characteristics have been evaluated in terms of polarity, selectivity, and separation efficiencies. These parameters were used to assess the outer silica surface contributions and the degree of surface deactivation brought about by different treatment techniques. The parent silica elutes the paraffinic hydrocarbons with high efficiency of separation and elutes aromatic hydrocarbons with nearly good separation and has bad separation of alcohols. The calcined silica at 500°C and 1000°C has a pronounced effect on the separation of aromatic hydrocarbons compared with the parent silica and hydrothermal treatment of silica. With respect to alcohols separation, the obtained bad separations using treated and untreated silica reflect the little effect of the thermal and hydrothermal treatment on the silica surface deactivation.

## 1. Introduction

 Gas chromatography (GC) is now widely used to examine the physicochemical characteristics of many solid and liquid materials. Carefully selected test solutes are injected into the flow of carrier gas and transported over the surface of stationary phase such as silica or others. The retention time and the peak elution profiles of standard solutes affected by interactions between the solutes and stationary phase are used to estimate those interactions [1, 2].

GC can be used for studying the behavior of stationary phase through investigating the interactions between these stationary phases and some solutes from different families [3–5]. 

Silica gels have long attracted attention since they are widely used and as stationary phase in chromatography, catalyst, extract, filter surface properties covering a wide range of acidity surface reactivity and pore structure [6–8]. The surface of silica gel can be modified easily by physical or chemical treatments leading in each case to different adsorptive properties. These treatments may include calcinations [9], hydrothermal treatment [10], silylation [11–13], and incorporation of organic and inorganic salts or organic compounds [14–17].

Usually, the separation of the mixture requires selective stationary phase, so there are many thousands of these phases, and the novelty of this approach is the silica gel preparation and its treatment with different methods in order to obtain different silica surfaces to cover most chromatographic problems. The purpose of this work is the synthesis and modification of silica gel through thermal and hydrothermal treatment to produce various silica surface modifiers as solid stationary phases in gas chromatography and their application to separate polar and nonpolar organic petroleum compounds.

## 2. Experimental

### 2.1. Preparation of Silica Gel

 Wide porous silica gel as a parent material was prepared by a conventional precipitation method according to the De Boer method [18]. The prepared silica gel was crushed and sieved using an automatic mechanical sieve. The portion from 60–80 mesh was selected, washed by 6 mol HCl, and stored for different physical and chemical treatments.

### 2.2. Thermal and Hydrothermal Treatments of Silica Gel

 The acid-washed silica gel (60–80 mesh) was subjected to various treatments, namely, thermal treatment and hydrothermal treatment, with the aim of producing different surface-modified silica gels. Thermal treatments were carried out by calcinations in air for 5 h at 1000°C. Hydrothermal treatments were carried out by reaction with liquid water at 220°C and 15 atm for 24 h in an autoclave. In addition, silica gels were obtained by a combination of two modifications in order to improve their properties as solid gas chromatographic stationary phases. The studied silica samples obtained using thermal and hydrothermal treatments are listed in Table 1.

### 2.3. Gas Chromatography

 The untreated silica and surface-modified silica gels were subjected to an inverse gas chromatography with the aim of investigating their performance as solid stationary phases. The solid material was packed in a stainless steel column (1/8 inch and 7 feet) by charging under vacuum. The packed column was activated at 200°C for 24 h in a stream of pure nitrogen gas at a flow rate of 15 mL min.

 The gas chromatograph used was an AT Unicam-610 equipped with FID. Nitrogen gas was used as a carrier at a flow rate of 25 mL min^−1^. The analysis was carried out at different temperatures depending on the column efficiency resulting in the optimum separation of the studied solutes. Different solutes, namely, mixtures of n-paraffins, aromatic and polyaromatic hydrocarbons, ketones, acetates, ethers, and n-alcohols were used as probes for the chromatographic characterization.

## 3. Results and Discussion

### 3.1. Rohrschneider Scheme

 Characterization of silica modified stationary phases according to Rohrschneider's method is based on the determination of retention indices for five solutes, namely, benzene, ethanol, methyl ethyl ketone, nitro methane, and pyridine on SE-30 (nonpolar stationary phase) and then on the solid stationary phases to be characterized. The retention index differences (Δ*I*) at 100°C can be calculated and then the so-called Rohrschneider constants Δ*I* X (Benzene), Δ*I* Y (Ethanol), Δ*I* Z (Methyl ethyl ketone), Δ*I* U (Nitro methane), and Δ*I* S (Pyridine) are obtained in Table 2. The elution order of the Rohrschneider probes is the same for all the studied silica-modified samples. The polarity of the chromatographic columns depends not only on the solid stationary phases under study but also on the solutes to be analyzed. Thus, each solute of the five solutes selected for this scheme of characterization may refer to a certain type of interaction between stationary phases and solutes.

 The retention indices of the modified silica (Si_C500_, Si_th_, and Si_Cth_) are given in Table 2, it has been found that Δ*I* of all modified silica below that of the untreated silica sample except the case of using ethanol and methyl ethyl ketone as props. The higher polarity of these excepted silica columns was focused, suggesting that the hydrothermal treatment of both untreated and calcined silica is nonselective stationary phases for polar compounds like alcohols and ketones. The ordered classification of stationary phases with numerical data is very valuable for comparison of columns for a particular type of analysis. The characterization reflects the deactivation degree of silica gel after modifications and the selectivity of these silica samples toward separation of solutes from different families.

### 3.2. Separation Efficiencies

**Figure 1:**
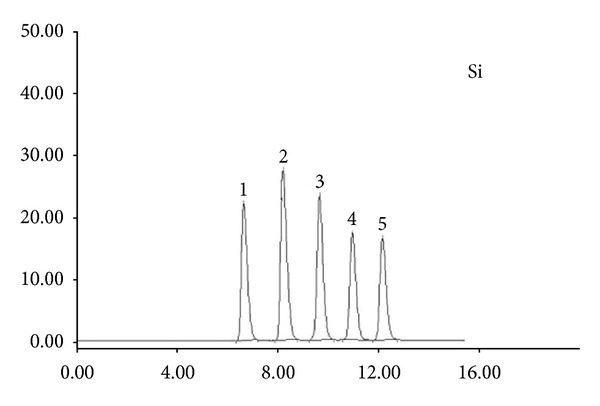
Separation of paraffinic hydrocarbons on parent silica 1-n-C_6_, 2-n-C_7_, 3-n-C_8_, 4-n-C_9_, and 5-n-C_10_.

**Figure 2:**
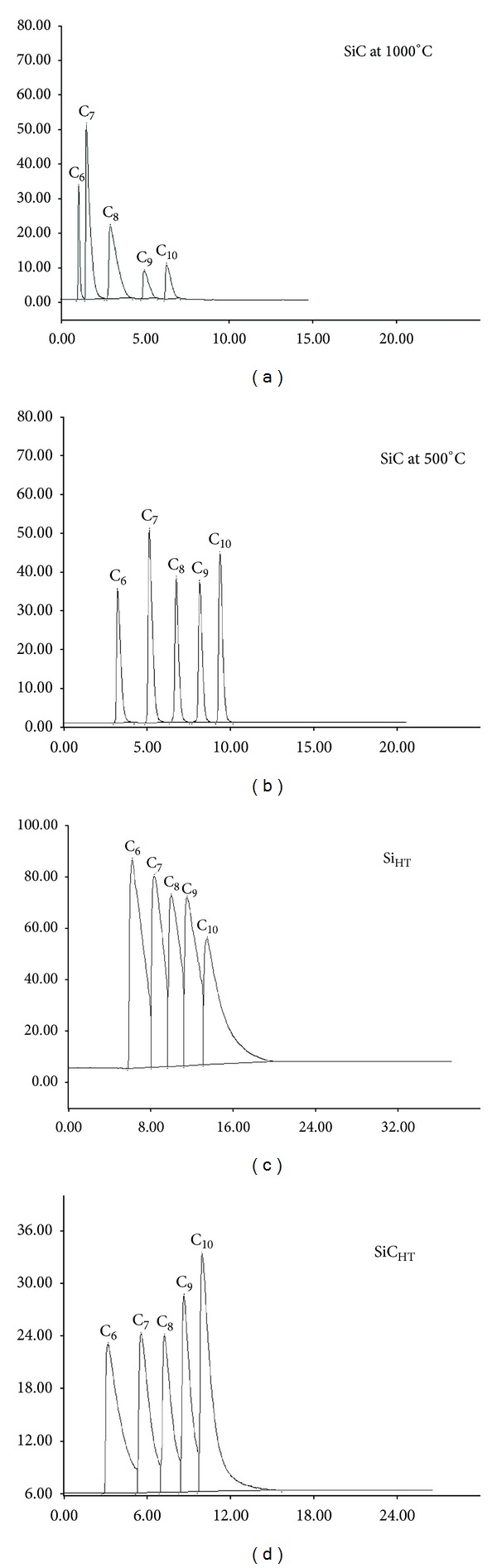
Separation of paraffinic hydrocarbons on modified silica gels 1-n-C_6_, 2-n-C_7_, 3-n-C_8_, 4-n-C_9_, and 5-n-C_10_.

**Figure 3:**
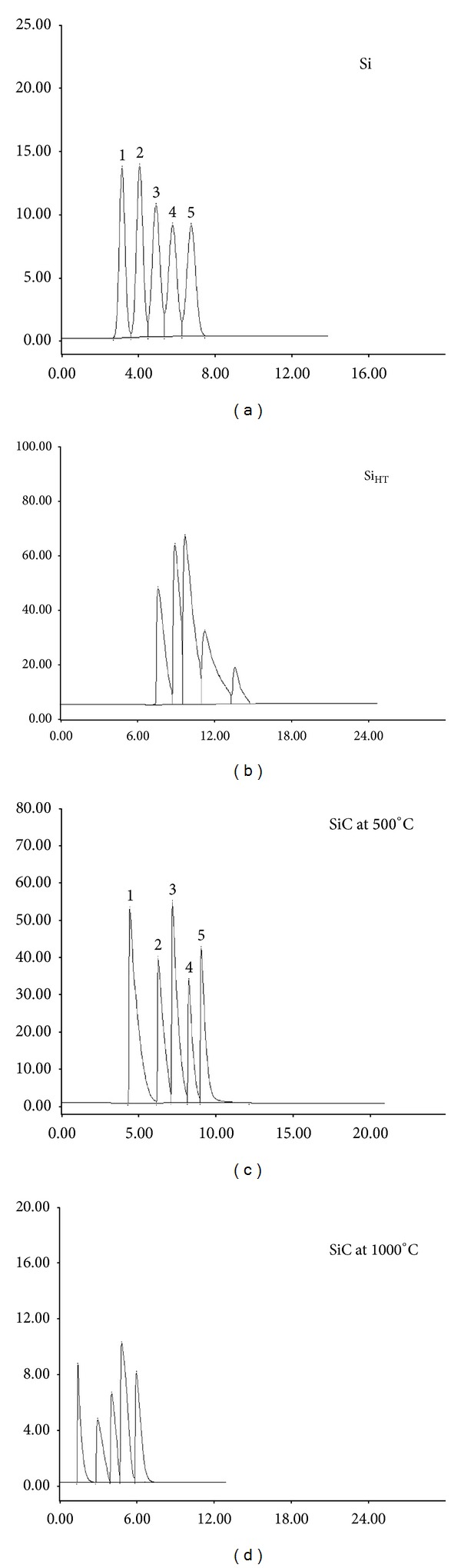
Separation of aromatic hydrocarbons on parent and modified silica gels (1-benzene, 2-toluene, 3-ethylbenzene, 4-propylbenzene, and 5-butylbenzene).

 The efficiency of gas chromatographic separation for the studied silica samples can be evaluated in terms of resolution and separation factor; and given in Table 3. The selected solutes represent their corresponding families; the solute pairs were chosen as test samples for evaluation of the selectivity of the untreated and treated silica gels as solid stationary phases in GC. The resolution and separation factor of Si_C500_ and Si columns show the highest values than the other columns using normal paraffins and aromatic hydrocarbons as props. This increases their separation efficiency and selectivity toward this type of analysis as shown in Figures 1–3. 

 For separation of alcohols, the parent and all modification techniques unsuccessful in this type of analysis and their resolution and separation factor are not available. These reflected on the alcohol separation giving bad elution accompanied with overlap and tailing peaks.

### 3.3. Selectivity and Applications

 The selectivity of the studied silica samples can be verified by using the uniformity criterion (Δ) for model systems comprising normal paraffines, aromatic and polyaromatic hydrocarbons, ethers, ketones, acetate, and alcohols. Consider

(1) $$\begin{array}{c} \Delta =\frac{nkt\;\text{keff}}{\tau }, \end{array}$$

where *nk* is the number of peaks on the chromatogram, *t* is the base width of the narrowest peak keff is the separation factor for the worst separated pair of components, and *τ* is the duration of analysis. From Table 4 and in Figures 1, 2, and 3, it has been found that all modifications differ in their separation efficiency according to the separated solutes. The saturated hydrocarbons can be separated as shown in Figures 1 and 2 with high efficiency by the parent and calcined silica at 500°C (Δ = 0.450 and Δ = 0.403, resp., for paraffins) compared with the calcined silica at 1000°C and hydrothermal treatment of silica.

 Also, the calcined silica at 500°C (Δ = 0.357) is the most efficient column for separation of aromatic hydrocarbons followed by the parent silica (Δ = 0.300) as shown in Figure 3. 

**Figure 4:**
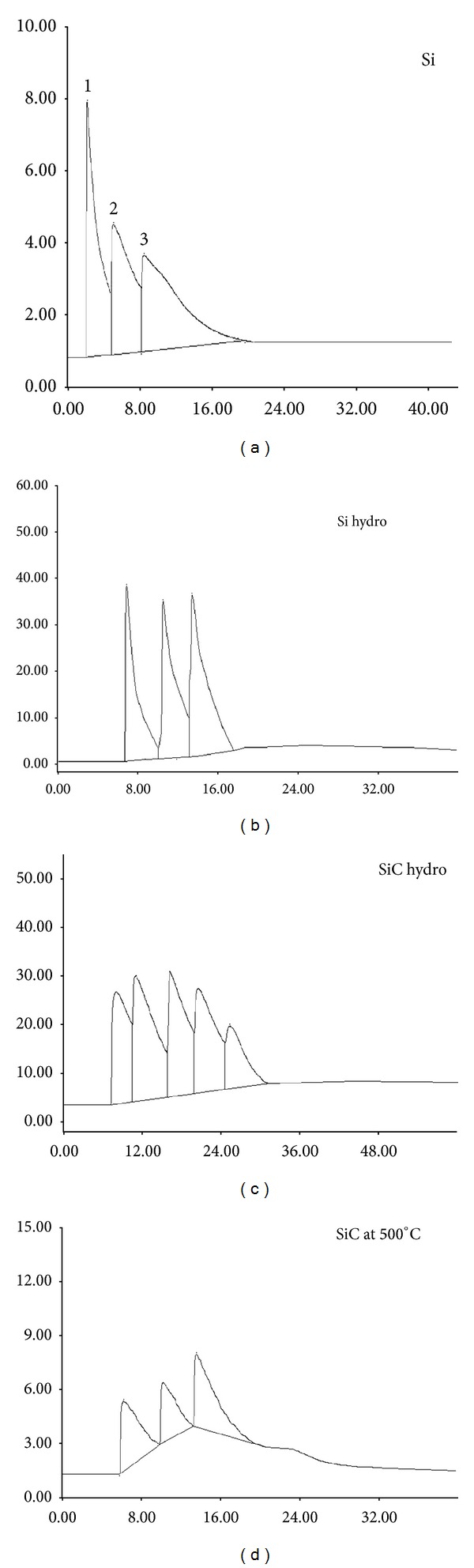
Separation of alcohols on parent and modified silica gels (1-C_3_-OH, 2-C_4_-OH, 3-C_6_-OH, 4-C_8_-OH, 5-C_10_-OH, and 6-C_12_-OH).

 For separation of alcohols, the parent and modified silica stationary phases failed in eluting alcohols which may be due to the fact that these treatment are not enough for deactivating the silica surface to a degree enough to separate the alcohol compounds as shown in Figure 4.

## 4. Conclusions

1. The resolution and separation factor of Si_C500_ and Si columns show the highest values compared other columns using normal paraffines and aromatic hydrocarbons as props. This increases their separation efficiency and selectivity toward this type of analysis. These were reflected by the highest average Rohrschneider props on parent silica than the other treatments.
2. The untreated silica gives suitable surface enough for eluting paraffinic hydrocarbons as previous works. The calcined silica at 500°C exhibits higher resolution values 3.918 for normal paraffins, which was confirmed with good separation of paraffines (Figure 1) giving symmetric and sharp peaks.
3. The aromatic hydrocarbons give nearly the same behavior of paraffines. The resolution of aromatic hydrocarbons on calcined silica at 500°C and Si columns shows higher values than the other columns.
4. The parent and treated silica gels failed in the separation of alcohols; this may be due to the fact that these treatments have little effect on the silica surface deactivation.
5. All studied parameters of characterization of silica gels via gas chromatography may be used in interpreting the reactivity of silica surfaces and factors controlling the adsorption of solutes.

## Figures and Tables

**Table 1:** List of the studied surface modified silica gels obtained by different techniques.

Sample no.	Notation	Treatment
1	Si	Acid-washed silica gel (60–80 mesh)
2	Si_C500_	Silica gel calcined at 500°C
3	Si_C1000_	Silica gel calcined at 1000°C
4	Si_HT_	Hydrothermally treated silica gel
5	Si_CHT_	Calcined silica gel followed by hydrothermal treatment

**Table 2:** Rohrschneider index for the parent and treated silica gels as solid stationary phases.

Column		Benzene (X)	Ethanol (Y)	Methyl ethyl ketone (Z)	Nitro methane (U)	Pyridine (S)	Average
Si	Δ*I*	144	602	720	418	830	542.8
Si_C500_	Δ*I*	49	584	681	327	763	480.8
Si_C1000_	Δ*I*	138	578	556	392	497	432.2
Si_HT_	Δ*I*	128	402	526	237	925	518.8
Si_CHT_	Δ*I*	93	606	847	387	733	533.2

**Table 3:** Separation factor and resolution of some solute pairs on the untreated and modified silica samples.

Column	Paraffin		Aromatic		Alcohol	
	C_6_, C_7_		Benzene, toluene		C_6_-OH, C_8_-OH	
	*α*	*R*	*α*	*R*	*α*	*R*
Si	1.237	3.132	1.183	2.732	NA	NA
Si_C500_	1.580	3.918	1.41	2.110	NA	NA
Si_C1000_	1.029	1.286	1.097	2.050	NA	NA
Si_HT_	1.081	NA	1.070	1.070	NA	NA
Si_CHT_	1.047	1.536	1.010	1.220	NA	NA

**Table 4:** Uniformity criterion for studied columns.

Column	Uniformity criterion			
	Paraffin	Aromatic	Polyaromatic	Alcohols
Si	0.450	0.300	0.270	—
Si_C500_	0.403	0.357	0.250	—
Si_C_	0.161	0.270	0.240	—
Si_HT_	—	0.182	0.166	—
Si_CHT_	0.400	0.210	0.210	—
